# Everybody's Page

**Published:** 1889-12-14

**Authors:** 


### Everybody's Page.

Icthyol collodion applied locally is reported to be very effectual in conjunction with the usual internal remedies in arresting the course of erysipelas.

The seventh edition of the Austrian Pharmacopœia is to come into use on the 1st January next. The last edition was published in 1869. The names of preparations in use in foreign pharmacopœias form a useful addition to the book.

Dr. W. Burck's pamphlet on the coffee plant disease is opportune just when the rise in price is considerable. The disease appears to be produced by a mould plant in the tissue of the coffee leaf which destroys the chief vital organs of the plant and causes the leaves to drop off.

Colonel Conway Gordon, in his report on the railways of India, states that the outbreak of scurvy at Singareni, in the Deccan, has disappeared, chiefly owing to the medical officer having adopted a system of supplying the workpeople daily with green vegetables from his garden. A hospital has been opened there.

The beneficial action of sea air on scrofula has been acknowledged and taken advantage of for centuries, but Dr. G. Badaloni, the Italian physician, has recently paid much attention to scrofulous affections, and he is able to show that the Italian hospices at Palermo, Fano, and Venice have been more successful in their treatment than foreign institutions of a similar nature.

Professor Berge, the eminent Belgian, is stated to have come to the conclusion that it is possible to determine accurately the quantity of nutritive elements indispensable to repair the working-man's physical powers, and consequently to determine how much money will be required in order to purchase the desirable quantity of nutrition. In other words, the employer of labour will be enabled, through the assistance of the chemist, to fix the minimum of wage with scientific accuracy. It is rather astounding to be told that a scientific man makes the egregious error of placing all men in one mould ; this is the work of the socialist faddist, rather than of the apostle of science. We cannot all exist on the same amount of food, however much we might wish to.

Perhaps some kind professor will come forward and determine, with scientific accuracy, the quantity of clothing indispensable to the covering of the human body. But, then, what will happen to that time-honoured custom in the City of selecting the gifts of the "livery cloth"? At present the Town Clerk has a much better time of it in cold weather than the Lord Chancellor or the Lord Chief Justice, for he receives six yards of black and six yards of green cloth wherewith to swathe his shivering limbs, whereas the great legal luminaries have to starve in four and a half yards of the best black cloth. Possibly the quality of the latter makes up for the short measure.

Candles, according to an American sketch, entitled "School Board Essays," are long thin things, like a white-washed ruler, with a piece of twine hanging out at one end. They are softer than a ruler, but harder than damp garden worms. They are of various sizes and smells, but they are nearly all greasy. Candles, like the opinions of members of Parliament, are to be had for money, but the candles are more durable, even if they are lighted in a draught. They are used for a variety of purposes. They hold up a window when one of the cords is broken, they bend sideways in glass bottles in shops. They are useful in bruises and when the hands are chapped. They thicken soup and give a flavour to gravy. They are of great value in greasing a pan to fry cabbage.

It is alleged that the germ of dry rot, which some botanists consider contagious, may be communicated to sound wood by tools which have been used in affected timber. If this is so, carpenters should be as careful over the cleaning of their saws and chisels as surgeons are with instruments.

At the meeting of reformed thieves and their patrons in Little Wild-street last week, when the annual supper was given by the St. Giles's Christian Mission, it was stated that 4,850 of the 19,800 prisoners discharged from the metropolitan prisons had signed the temperance pledge. It would be interesting to know how many such men who annually sign the pledge conscientiously keep it.

A curious superstition said to exist in Brittany for many ages, and which possibly is still believed in, is that a child is protected from every disease by having its shirt put on damp. We can readily believe in the efficacy of such a drastic measure ; sudden death would doubtless speedily place the child beyond the power of the most fell disease. The very thought of a damp shirt at this time of the year makes cold water trickle down one's spine.

During the cold weather many people wear thick socks or stockings. The sooner those who suffer from chilblains or corns give them up the better for their comfort. Tight boots are generally supposed to be the great producers of corns, but loose boots and thick socks are equally objectionable. Few remedies allay the troublesome and at times almost maddening itching of chilblains better than soaking the part affected in hot water.

Hellebore, which is sometimes mixed with insect powder and which has by some people been looked upon as poisonous to flies, does not appear to add in any way to the destructive powers of the powders with which it has been mixed ; for if flies are placed under a bell jar with hellebore they do not seem to find it antagonistic to their health, as after remaining in confinement for twenty-four hours they are generally as merry as anyone could be expected to be under similar circumstances.

In the event of an outbreak of influenza in this country, by no means improbable, wise people will be most careful in having sufficient warm clothing and in avoiding a chill. The origin of such epidemics is obscure, but there are some people who believe that the lower animals are not altogether guiltless. It is, however, rather hard to add yet one more sin to the long list already attributed to the domestic cat. Let us draw a veil over the conflicting emotions of the maiden ladies whose devotion to grimalkin and fear of influenza must now be weighed in the scales.

By the way, why do we always speak of catching a cold, when, notwithstanding the perseverance we may display in our futile endeavours to avoid it, it invariably catches us and clings to us with an affection we consider cruelly misplaced?

It is stated that the use of lacquer for preserving ships' keels has been known in Japan for some time ; and according to the Japanese newspapers its efficiency for this purpose has, at length, been recognised by foreigners. A report has recently been published on the effect of the lacquering process by a Russian naval officer, from which it appears that the process was applied to his ship with great success. The lacquer was applied to the zinc as well to the steel portions of the vessel, but it was found that it peeled off the zinc sheathing in a few weeks ; on the other hand, an examination of the vessel's keel, after the lapse of a year, showed that the lacquer on the steel portion was so well preserved and hard that it could not be scraped off without great difficulty.

